# Characterization of complete mitochondrial genome of *Periclimenes brevicarpalis* (Decapoda: Palaemonidae)

**DOI:** 10.1080/23802359.2024.2417927

**Published:** 2024-10-18

**Authors:** Chao Peng, Sigang Fan, PengFei Peng

**Affiliations:** a Hunan Provincial Collaborative Innovation Center for Efficient and Health Production of Fisheries, Hunan University of Arts and Science, Hunan, Changde, China; b Key Laboratory of Aquatic Product Processing, Ministry of Agriculture, South China Sea Fisheries Research Institute, Chinese Academy of Fishery Sciences, Guangzhou, China; c South China Sea Marine Survey and Technology Center, State Oceanic Administration, Key Laboratory of Marine Environmental Survey Technology and Application, Ministry of Natural Resources, Guangzhou, China

**Keywords:** *Periclimenes brevicarpalis*, mitochondrial genome, phylogeny, Palaemonidae

## Abstract

*Periclimenes brevicarpalis* is widely distributed in the Indo West Pacific oceans. The mitochondrial genome of *P. brevicarpalis* was sequenced and assembled firstly by next generation sequencing technology in our study. The complete mitochondrial genome of* P. brevicarpalis* was 16,673 bp in size, consisted of 22 transfer RNA genes, 13 protein coding genes and two ribosomal RNA genes. The contents of the four bases were C (25.62%), T (28.73%), A (31.64%), and G (14.01%). The result of phylogenetic analysis showed that *P. brevicarpalis*was clustered with Anchistus australis. In conclusion, our research provided valuable data for phylogenetic analysis of the Palaemonidae family as the first report about mitochondrial genomes in Periclimenes.

## Introduction

The shrimp *Periclimenes brevicarpalis* (Schenkel, 1902) belongs to family Palaemonidae. Oceans Indian and Pacific are rich in this species (Bruce 1971). The appearance of this species is characterized by a transparent body. In addition, the chelipeds and pereiopods are blue, while the telson and uropods are brown with orange centers (Lee and Ko 2014). *P. brevicarpalis* and sea anemones have a symbiotic relationship (Sanjeevi et al. 2017).

The mitogenome will provide a valuable information for germplasm evaluation, genetic diversity, and phylogeography (Desalle et al. 2017). However, only partial COX1, 16S and 12S sequences of *P. brevicarpalis* are registered in GenBank. Limited genetic resources has hindered the implementation of effective conservation and utilization of *P. brevicarpalis*. The complete mitogenome of *P. brevicarpalis* was firstly described, which is also the first report for the genus *Periclimenes.* In addition, a phylogenetic analysis within Palaemonidae was also conducted.

## Materials and methods

**Figure 1.**
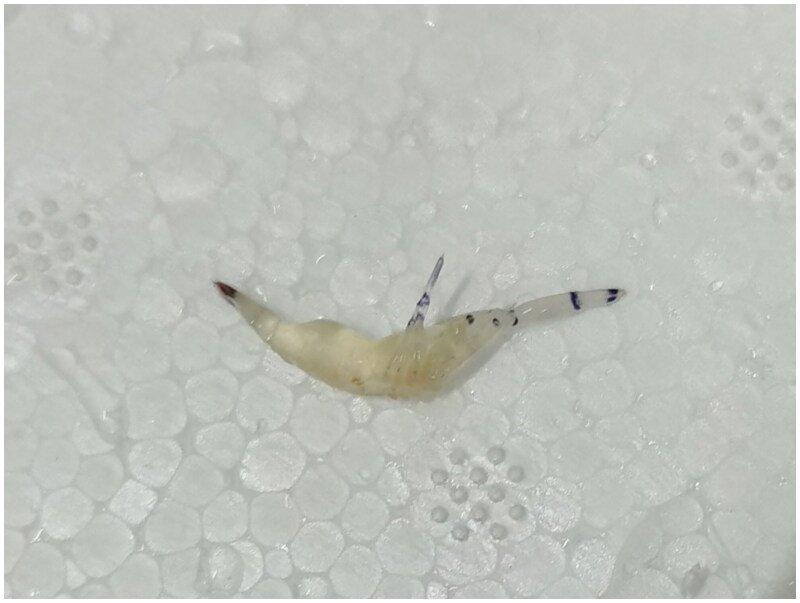
*Periclimenes brevicarpalis* (photo from Sigang Fan).

A shrimp (voucher no. SOA210322-1; Figure 1) was catched from the South China Sea (22°78′N, 115°12′E), Shanwei, Guangdong Province, China, and was deposited in the South China Sea Fisheries Research Institute (contact person: Sigang Fan, email: fansigang@scsfri.ac.cn). The morphology of the shrimp was same with *P. brevicarpalis* described by Lee and Ko (2014). In addition, the partial 16S (JX025191.1) and 12S (KJ019599.1) sequences of *P. brevicarpalis* had more than 99% identity with that of the shrimp. Therefore, the shrimp obtained by us should be *P. brevicarpalis.* The muscle tissues of *P. brevicarpalis* were scissored and deposited at −80 °C. Using the EasyPure^®^ Marine Animal Genomic DNA Kit (TransGen, Beijing, China), DNA was extracted from muscle tissue. The nextera XT DNA Library Prep kit (Illumina, San Diego, CA, USA) was used to prepare the libraries, and Illumina HiSeq 2000 was used for the sequencing. After removing the adapter sequences, Fastp 0.17.0 (https://github.com/OpenGene/fastp) was performed to delete low quality sequences (base quality ≤ 20) and poly-N, and then assembled raw data into clean data (Chen et al. 2018). The protein-coding genes (PCGs) were analyzed by ORF finder and MITOS (Bernt et al. 2013). rRNA and tRNA were identified using MITOS (Bernt et al. 2013). Compositional bias was analyzed by calculating AT and GC skews. The formulas were listed as followed: AT skew = (A − T)/(A + T) and GC skew = (G − C)/(G + C). OGDRAW program was use to draw a circular mitochondrial genome map (Greiner et al. 2019).

The phylogenetic analyses of 16 species of family Palaemonidae were conducted using 13 PCGs. All 16 species were listed as followed: *Periclimenes brevicarpalis*, *Exopalaemon carinicauda*, *Palaemon annandalei*, *E. modestus*, *P. gravieri*, *P. tenuidactylus*, *P. elegans*, *P. serenus*, *P. sinensis*, *Macrobrachium rosenbergii*, *M. bullatum*, *M. lanchesteri*, *M. nipponense*, *Hymenocera picta*, *Anchistus australis*, *Gnathophyllum americanum*, and *P. brevicarpalis. Panulirus stimpsoni* (GQ292768.1) (Liu and Cui 2011) was used as the outgroup. Phylogenetic analysis was performed using maximum-likelihood (ML) model with 1,000 bootstrap replications in MEGA X (Kumar et al. 2018).

## Results

**Figure 2.**
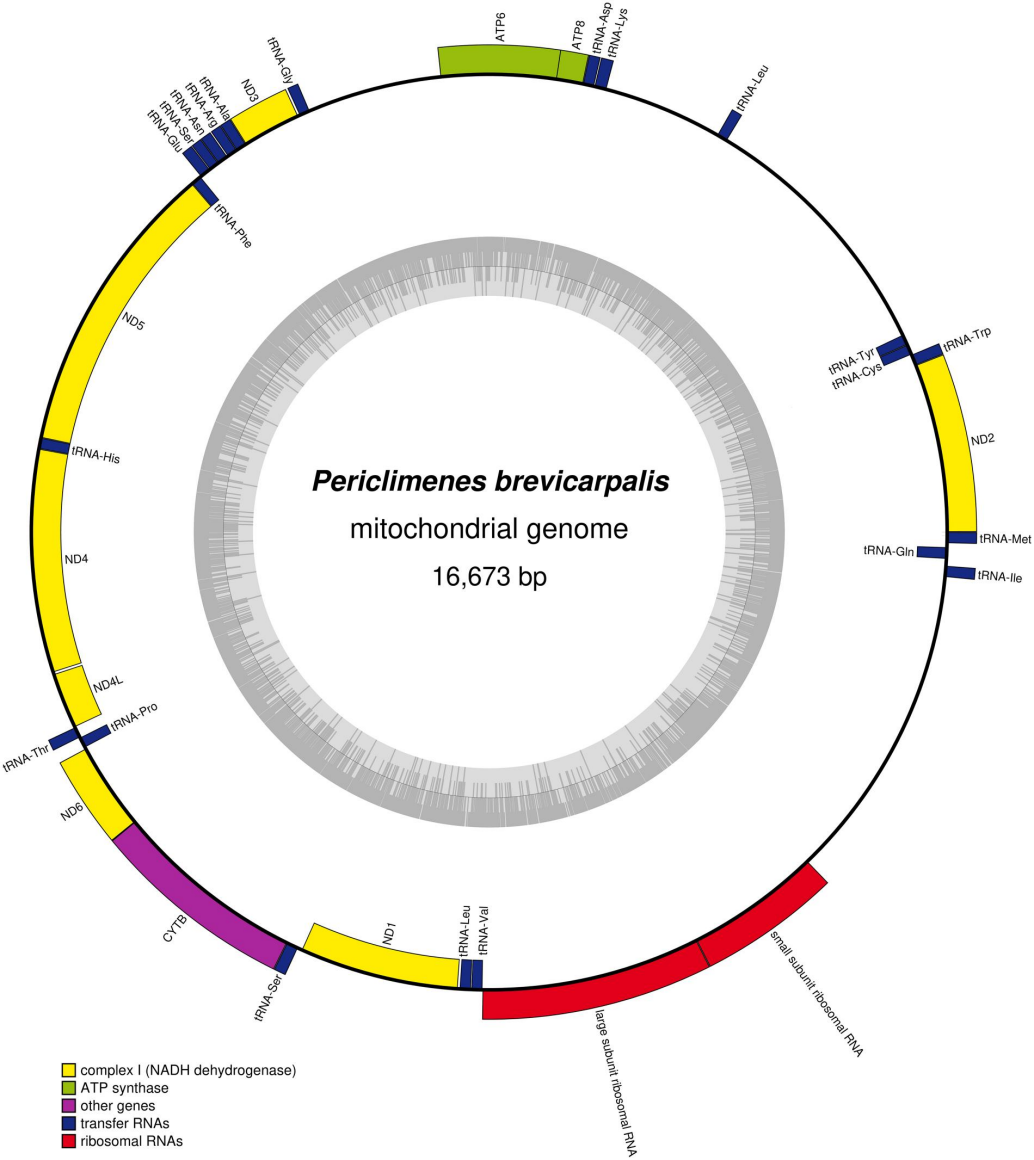
Mitochondrial genome map of the *P. brevicarpalis.*

**Figure 3.**
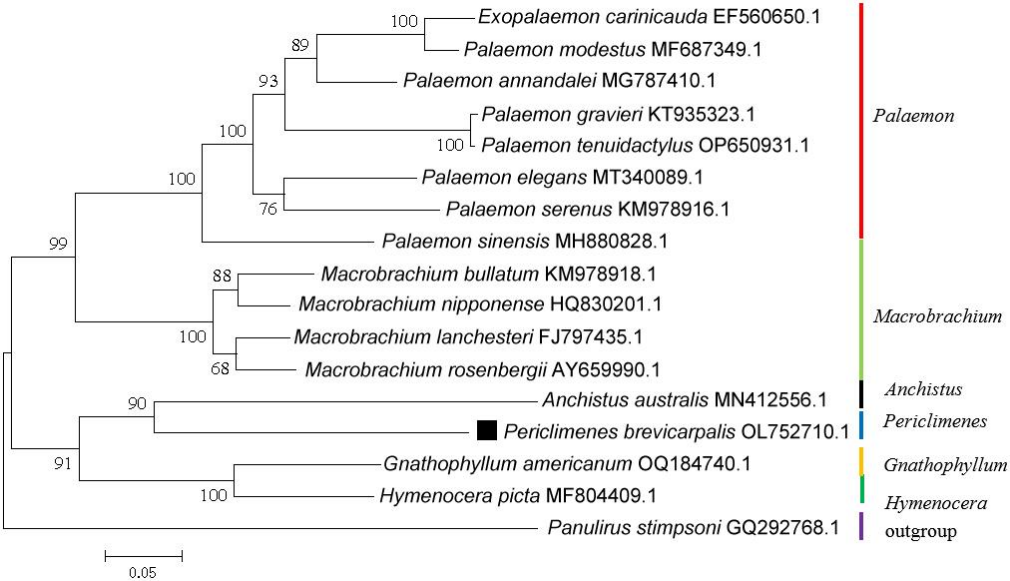
Phylogenetic tree of *P. brevicarpalis* and related species based on maximum-likelihood method. Species used include the following: *Exopalaemon carinicauda* (Shen et al. 2009), *Palaemon annandalei* (Yuan et al. 2018), *Exopalaemon modestus* (Wang et al. 2018), *Palaemon gravieri* (Kim et al. 2017), *Palaemon tenuidactylus*, *Palaemon elegans* (González-Castellano et al. 2020), *Palaemon serenus* (Gan et al. 2016), *palaemonetes sinensis* (Zhao et al. 2019), *Macrobrachium rosenbergii* (Miller et al. 2005), *Macrobrachium bullatum*, *Macrobrachium lanchesteri*, *Macrobrachium nipponense* (Ma et al. 2011), *Hymenocera picta* (Sung et al. 2018), *Anchistus australis* (Liu 2019), *Panulirus stimpsoni* (Liu and Cui 2011), *Gnathophyllum americanum* (Sung et al. 2023), and *Periclimenes brevicarpalis* (this study).

A total of 14.15 million raw sequencing reads were obtained. A total of 13.95 million clean reads were generated after quality filtering. The Q20 and Q30 of clean reads were 96.18% and 90.86%, respectively. The read coverage depth map of *P. brevicarpalis* is shown in Supplementary Figure S1. The complete mitochondrial genome of *P. brevicarpalis* was sequenced and submitted into GenBank under accession number OL752710.1. It was 16,673 bp in size. 37 mitochondrial genes including 13 PCGs, 22 tRNA genes and 2 rRNA genes were detected in mitogenome (Figure 2). Its overall base composition on the heavy strand was A: 31.64%, G: 14.01%, T: 28.73%, C: 25.62%. The light strand encoded four PCGs (ND1, ND4, ND4L, and ND5), and eight tRNA genes. Twenty-five genes were encoded by the heavy strand. Almost PCGs were initiated with ATN as the start codon except ND5 used GTG as the start codon. The complete termination codons of the PCGs were TAA (ND1, ND2, ND4L, ND6 and Cox1), TAG (ND3 and ATP8). Another six PCGs contained an incomplete T or TA stop codon. The AT and GC skewness of the mitogenome sequence were 0.048 and −0.2930, respectively. The total length of the 13 PCGs of *P. brevicarpalis* was 11,160 bp, which accounted for 66.93% of the complete mitogenome. 16S rRNA was 1,285 bp in size with 67.00% A + T content, and 12S rRNA was 793 bp in length (AT%=65.45%). Size of tRNA genes ranged from 61 to 71 bp in length. In order to analyze the evolutionary relationships of *P. brevicarpalis* in family Palaemonidae, the thirteen complete concatenated PCGs from other 15 shrimp belong to five genera were downloaded from GenBank database. The result of phylogenetic tree showed that *P. brevicarpalis* and *A. australis* were clustered together firstly (Figure 3). Species of the same genus were grouped together in a clade. *Periclimenes* and *Anchistus* were in sister relationship. The phylogenetic relationships among the six genus were similar with other studies (Zhao et al. 2019; González-Castellano et al. 2020).

## Discussion and conclusion

The length of *P. brevicarpalis* mitogenomes was 16,673 bp, longer than that of other Palaemoninae (González-Castellano et al. 2020; Sung et al. 2018; Sun et al. 2023). Same to most Palaemoninae, *P. brevicarpalis* mitogenome contained 2 rRNA, 13 PCGs, and 22 tRNAs genes (Zhao et al. 2019; González-Castellano et al. 2020). As previous research (Zhao et al. 2019; Sung et al. 2023), the most PCGs were started by an ATN codon except nad5. The incomplete stop codon (T or TA) was also detected in *P. Sinensis*, *E. annandalei* and *G. americanum* (Zhao et al. 2019; Yuan et al. 2018; Sung et al. 2023), which was consisted with our study. The result of AT and GC skew showed that the mitogenemo of *P. brevicarpalis* was the A-skew and C-skew. The AT skew of *P. brevicarpalis* was similar with *Palaemon capensis* (0.048) and *P. gravieri* (0.047), and lower than *Macrobrachium* species (0.100-0.157) (Zhao et al. 2019). In addition, the GC skew of *P. brevicarpalis* was consisted with that of other Palaemoninae mitochondrial genomes (Zhao et al. 2019). In this research, the evolutionary tree including many species from family Palaemonidae was constructed and analyzed. All nodes were strongly supported. The result of phylogenetic tree showed that *P. brevicarpalis* was clustered with *A. australis,* which was firstly reported (Figure 3). Consistently, this research showed that *Palaemon* and *Macrobrachium* were in sister relationship, which was also reported by another research (Zhao et al. 2019; González-Castellano et al. 2020). We also found that *Hymenocera* were sister genus to the genus *Gnathophyllum*, which was consisted with previous research (Sung et al. 2023).

In conclusion, the present study firstly sequenced, assembled and characterized the complete mitogenome of *P. brevicarpalis.* It also constructed the phylogenetic tree of Palaemonidae, which provide useful information for molecular evolution and conservation of this species.

## Supplementary Material

Supplemental Material

## Data Availability

The genome sequence data that support the findings are openly available in GenBank of NCBI (accession no. OL752710.1). The associated BioProject, SRA, and Bio-Sample numbers are PRJNA788149, SRR17206303, and SAMN23928634, respectively.
