# Supplementary figures and images for: Characterization of complete mitochondrial genome of Periclimenes brevicarpalis (Decapoda: Palaemonidae)
Source: Mitochondrial DNA B Resour. 2024 Oct 18;9(10):1414–7. doi: 10.1080/23802359.2024.2417927 (PMC11492385; doi:10.1080/23802359.2024.2417927)

# Sequencing Depth and Coverage Map

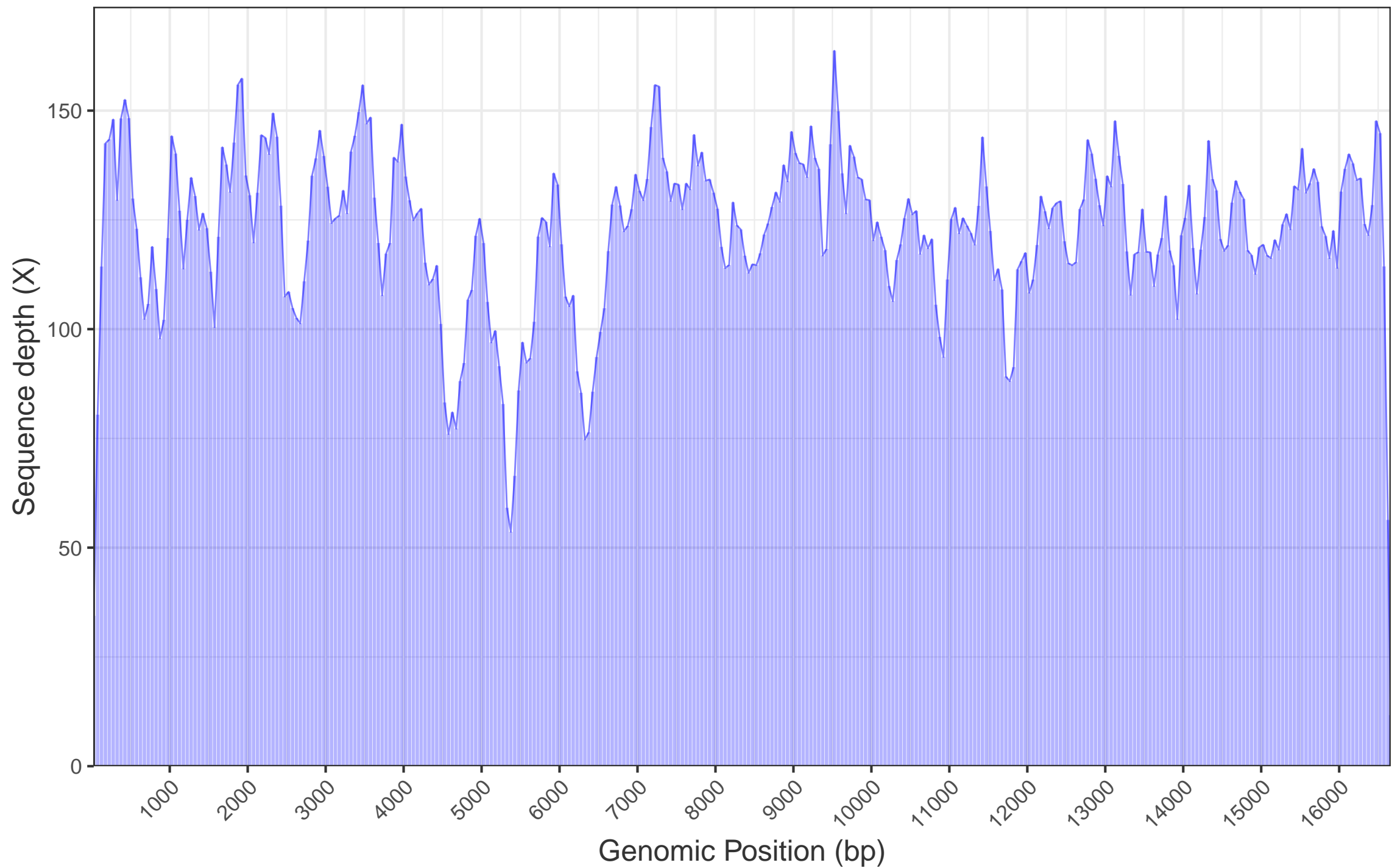

Supplement: Supplemental Material [file TMDN_A_2417927_SM8527.pdf]
